# Empagliflozin and Colchicine in Patients With Reduced Left Ventricular Ejection Fraction Following ST-Elevation Myocardial Infarction: A Systematic Review

**DOI:** 10.7759/cureus.92680

**Published:** 2025-09-18

**Authors:** Francis Asante Baadu, Muhammad Ahtsham Arif, Zara Riaz, Manoj Argariya, Karishma rani, Mubasshra Iqbal

**Affiliations:** 1 Internal Medicine, Komfo Anokye Teaching Hospital, Kumasi, GHA; 2 General Internal Medicine, Royal Blackburn Teaching Hospital, Blackburn, GBR; 3 Internal Medicine, University Hospitals Birmingham, Birmingham, GBR; 4 Internal Medicine, HBS Medical and Dental College, Islamabad, PAK; 5 Medicine, Rawal Institute of Health Sciences, Islamabad, PAK

**Keywords:** colchicine, empagliflozin, hospitalization, inflammation, myocardial infarction, treatment outcome, ventricular dysfunction

## Abstract

Patients with reduced left ventricular ejection fraction (LVEF) following ST-elevation myocardial infarction (STEMI) are at a high risk for heart failure (HF), adverse ventricular remodeling, and cardiovascular mortality. Empagliflozin, a sodium-glucose co-transporter 2 (SGLT2) inhibitor, and colchicine, an anti-inflammatory agent, have been proposed as adjunctive therapies to mitigate post-myocardial infarction (MI) complications. A systematic review of randomized controlled trials (RCTs) published in English between January 2015 and June 2025 was conducted using PubMed, Embase, and Cochrane Library. Fourteen RCTs were included. Risk of bias assessment using Cochrane RoB 2.0 classified five studies as low risk and eight as of some concern. Evaluating empagliflozin or colchicine in post-STEMI patients with LVEF <45% was conducted. Key outcomes included changes in LVEF, N-terminal pro-B-type natriuretic peptide (NT-proBNP), HF hospitalization, mortality, inflammatory biomarkers (CRP, creatine kinase-myocardial band (CK-MB)), and safety profiles. The Cochrane Risk of Bias 2.0 was used for quality assessment, and data were synthesized thematically. Visual summaries were generated using the robvis tool. The review indicated that empagliflozin consistently improved LVEF by 1.5%-5.7%, reduced NT-proBNP by approximately 15%, and lowered HF hospitalization risk, though it did not significantly reduce all-cause mortality. Colchicine reduced inflammatory markers (CRP, CK-MB) and recurrent ischemic events, but its effects on LVEF and HF outcomes were inconsistent. Colchicine was also associated with a higher incidence of gastrointestinal side effects and an increased risk of left ventricular thrombus formation. Empagliflozin showed consistent benefits in improving ventricular function and reducing HF hospitalizations after STEMI in patients with type 2 diabetes and should be prioritized in this setting. Colchicine provided potential anti-inflammatory and ischemic protection but demonstrates inconsistent cardiovascular efficacy and notable tolerability concerns. Further high-quality RCTs are required to clarify its role.

## Introduction and background

ST-elevation myocardial infarction (STEMI) remains one of the most serious manifestations of coronary artery disease and is a leading cause of death worldwide [1]. Despite advances in timely reperfusion therapies such as percutaneous coronary intervention (PCI), a substantial proportion of patients experience persistent left ventricular dysfunction and reduced left ventricular ejection fraction (LVEF) [2]. This impaired cardiac function not only predisposes patients to heart failure (HF) but also contributes to high long-term morbidity and mortality [3]. Current standard therapies focus on restoring blood flow and reducing recurrent ischemic events, yet effective strategies to prevent post-infarction remodeling and progressive HF remain limited [3].

Among the emerging therapeutic options, two agents have attracted significant attention. Empagliflozin, a sodium-glucose cotransporter 2 (SGLT2) inhibitor originally developed for type 2 diabetes, has shown consistent cardiovascular benefits in patients with heart failure with reduced ejection fraction (HFrEF) [4]. Its proposed mechanisms include improved myocardial energetics, diuretic and hemodynamic effects, and reductions in fibrosis and inflammation [5,6]. Colchicine, an established anti-inflammatory medication, has demonstrated cardiovascular protective effects through inhibition of the Nod-like Receptor family pyrin domain containing 3 (NLRP3) inflammasome and suppression of pro-inflammatory cytokine signaling, thereby limiting myocardial injury and adverse remodeling after an infarction [7,8]. Low-dose colchicine has further been shown to reduce recurrent ischemic events and attenuate neutrophil-driven inflammation and fibrosis in patients with coronary artery disease [9,10].

While both drugs have individually shown promise in cardiovascular disease, their comparative effectiveness in the specific context of STEMI patients with reduced LVEF has not been systematically evaluated [11]. Previous systematic reviews have primarily focused on SGLT2 inhibitors or colchicine separately in cardiovascular disease, but none have synthesized evidence that directly contrasts their roles in post-STEMI recovery [12]. Given the ongoing burden of HF and poor outcomes in this population, there is a need to evaluate how these agents compare in terms of safety and effectiveness [13].

This systematic review seeks to answer the question: Do empagliflozin and colchicine improve outcomes in patients with STEMI who develop reduced LVEF, and how do their effects compare? We hypothesize that both agents provide beneficial effects after STEMI, though through distinct mechanisms: empagliflozin by enhancing hemodynamic and metabolic recovery, and colchicine by attenuating inflammation and fibrosis. Clarifying these differences will help inform therapeutic decision-making and highlight areas for future research.

## Review

Methodology

The review adhered to Preferred Reporting Items for Systematic Reviews and Meta-Analyses (PRISMA) guidelines and employed the PICO framework to formulate the research question, as outlined in Table 1 [14,15].

**Table 1:** PICO framework

Concepts	Text words	Controlled vocabulary (MeSH Terms)
Population/Problem	“Post-STEMI” OR “ST-Elevation Myocardial Infarction” OR “Acute Myocardial Infarction” OR “Reduced LVEF”	"Myocardial Infarction" [Mesh] OR "ST Elevation Myocardial Infarction" [Mesh] OR "Ventricular Dysfunction, Left" [Mesh]
Intervention	“Empagliflozin”	"Empagliflozin"
Comparison	“Colchicine”	"Colchicine" [Mesh]
Outcome	“Heart failure hospitalization” OR “LVEF improvement” OR “Cardiovascular death” OR “Cardiac remodeling” OR “NT-proBNP reduction”	"Heart Failure" [Mesh] OR "Hospitalization" [Mesh] OR "Ventricular Remodeling" [Mesh] OR "Ejection Fraction" [Mesh] OR "Brain Natriuretic Peptide, N-Terminal" [Mesh] OR "Mortality" [Mesh]

The population was defined as patients with STEMI and reduced LVEF (≤40%), the interventions were empagliflozin or colchicine individually compared with placebo or standard therapy, and the outcomes included HF hospitalization, adverse cardiac remodeling, mortality, and related endpoints. The literature search was conducted across PubMed, Embase, and the Cochrane Library, and the Boolean operator “OR” was used between empagliflozin and colchicine to ensure inclusion of studies evaluating either drug separately. The search was limited to 2015-2025, reflecting the era when both agents began systematic evaluation in large-scale cardiovascular outcome trials relevant to contemporary practice. Although the protocol was not prospectively registered in International Prospective Register of Systematic Reviews (PROSPERO), which is acknowledged as a limitation, the review process maintained transparency and rigor. Study selection and data extraction were conducted independently by two reviewers using predefined criteria, with disagreements resolved through discussion and arbitration by a third reviewer when necessary. Risk of bias was similarly assessed by two independent reviewers using the Cochrane RoB 2.0 tool, with consensus achieved through discussion or third-party adjudication.

Search strategy and search string

Electronic databases searched included PubMed, Embase, and Cochrane. Searches were systematically performed using MeSH terms. Boolean operators "AND" and "OR" were applied to appropriately link search terms and ensure broad and precise coverage of the literature. search string followed the (("Myocardial Infarction"[Mesh] OR "ST Elevation Myocardial Infarction"[Mesh] OR "Ventricular Dysfunction, Left"[Mesh]) AND ("Empagliflozin"[Mesh] OR "Colchicine"[Mesh])) AND ("Heart Failure"[Mesh] OR "Hospitalization"[Mesh] OR "Ventricular Remodeling"[Mesh] OR "Ejection Fraction"[Mesh] OR "Mortality"[Mesh]). Although the initial search string combined both terms (empagliflozin AND colchicine), the included studies evaluated the drugs separately rather than in combination. Accordingly, the final analysis considered randomized controlled trials (RCTs) of either empagliflozin or colchicine individually against placebo or standard care.

Studies selection criteria

Inclusion Criteria

The population included patients with STEMI and reduced LVEF (≤40%), defined in accordance with European Society of Cardiology (ESC) and American Heart Association (AHA) guidelines. Eligible studies were RCTs that investigated empagliflozin or colchicine individually, each compared with placebo or standard therapy. Studies were required to report at least one clinically relevant outcome, including HF hospitalization, cardiac remodeling, mortality, or related cardiovascular endpoints. Only full-text articles published in English between 2015 and 2025 were considered, reflecting the era when both agents began systematic evaluation in large-scale cardiovascular outcome trials.

Exclusion Criteria

Studies were excluded if they were non-randomized, observational, case reports, reviews, editorials, or preclinical (animal/in vitro) investigations. Trials were also excluded if they did not involve patients with MI, did not clearly report outcomes related to HF or cardiovascular endpoints, or focused on empagliflozin or colchicine for non-cardiovascular indications (e.g., diabetes management alone, pericarditis, or gout).

Study selection process

The study selection was conducted in a two-stage process. First, the titles and abstracts of all the retrieved records were screened independently by two reviewers to identify potentially eligible studies. In the second stage, the full texts of the shortlisted articles were assessed in detail against the predefined inclusion and exclusion criteria. Discrepancies between the two reviewers were resolved through discussion, and when consensus could not be reached, a third reviewer acted as an arbitrator. Only articles that fulfilled all inclusion criteria were selected for the final synthesis. The review was limited to manuscripts published between 2015 and 2025, reflecting the period during which both empagliflozin and colchicine began systematic evaluation in cardiovascular outcome trials [16].

Methodological quality assessment

The Cochrane Risk of Bias 2.0 (RoB 2.0) was used as the methodological quality measure of the included RCTs, which divided the studies in terms of low risk, some concerns, or high risk of bias due to their bias regarding multiple domains [17]. Visual summaries were generated using the robvis tool [18].

Data Extraction and Synthesis

The data extract form was designed to organize in a standardized form the following details in each study: author/year, study design, sample size, baseline patient demographics, initial LVEF values, details of the intervention, primary outcomes, secondary outcomes, adverse events and key findings. In order to recognize the common patterns, a thematic synthesis was employed following an inductive, data-driven approach and formulating a comparative understanding of and among trials [19].

Ethical Consideration

**Figure 1:**
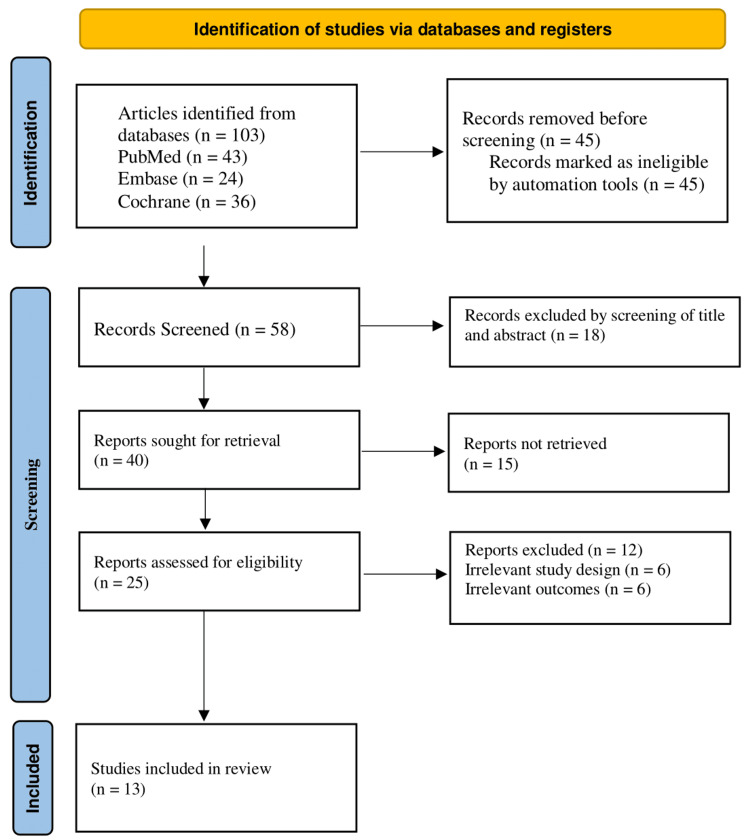
PRISMA flowchart PRISMA: Preferred Reporting Items for Systematic Reviews and Meta-Analyses.

The review adhered to the Declaration of Helsinki, followed PRISMA recommendations, and was transparent, reproducible, and methodologically sound. It supported evidence-based clinical decision-making by publishing findings in peer-reviewed journals (Figure 1).

Results

A total of 103 articles were initially identified through database searches, including 43 from PubMed, 24 from Embase, and 36 from the Cochrane Library. Before screening began, 45 records were removed because they were marked as ineligible by the automation tools. This left 58 records to be screened based on titles and abstracts. Following this screening process, 18 records were excluded. The remaining 40 reports were sought for retrieval. However, 15 of these could not be retrieved, leaving 25 reports to be assessed for eligibility. Of these, 12 reports were excluded due to irrelevant study design (n=6) or irrelevant outcomes (n=6). Ultimately, 13 studies met all the inclusion criteria and were included in the final review.

Cochrane Risk of Bias Assessment

Table 2 in the Cochrane systematic review assesses the risk of bias in studies, indicating high, low, or some concern based on selection, performance, detection, and reporting methods.

**Table 2:** Cochrane Risk of Bias assessment [20-32]

Study ID	Bias from randomization process	Bias due to deviations from intended interventions	Bias due to missing outcome data	Bias in measurement of the outcome	Bias in selection of the reported result	Overall risk of bias
Carberry et al., 2025	Low	Low	Low	Low	Low	Low
Butler et al., 2024	Low	Low	Low	Low	Low	Low
Jolly et al., 2025	Low	Low	Low	Low	Low	Low
Tong et al., 2020	Low	Low	Low	Low	Low	Low
von Lewinski et al., 2022	Low	Low	Low	Low	Low	Low
Tardif et al., 2019	Low	Some concerns	Low	Low	Low	Moderate
Bouleti et al., 2024	Low	Some concerns	Low	Low	Low	Moderate
Karim et al., 2025	Low	Low	Low	Some concerns	Low	Moderate
Giannopoulos et al., 2015	Some concerns	Low	Some concerns	Low	Low	Moderate
Hernandez et al., 2024	Low	Low	Low	Low	Some concerns	Moderate
Udell et al., 2024	Low	Some concerns	Low	Low	Some concerns	Moderate
Sourij et al., 2024	Some concerns	Low	Low	Low	Low	Moderate
Mewton et al., 2021	Low	Low	Low	Low	Some concerns	Moderate

Based on the Cochrane RoB 2.0 assessment, five studies had a low risk of bias, and eight studies had a moderate risk of bias.

Overview of Included Studies and Key Findings

Table 3 details the study characteristics and findings of studies included in the systematic review.

**Table 3:** Overview of the included studies and their key findings *p<0.05, indicating statistically significant improvements; LVEF: Left ventricular ejection fraction; LVEDVI: Left Ventricular End-Diastolic Volume Index; LAVI: Left Atrial Volume Index; LVMI: Left Ventricular Mass Index; NT-proBNP: N-terminal pro-B-type natriuretic peptide; hs-TnI: high-sensitivity troponin I; LVESVI: Left Ventricular End-Systolic Volume Indexed to Body Surface Area; Left Ventricular End-Systolic Volume Index; HF: Heart Failure; MI: Myocardial Infarction; HR: Hazard Ratio; MRI-LGE: Magnetic Resonance Imaging – Late Gadolinium Enhancement; IQR: interquartile range; CK-MB: Creatine Kinase-Myocardial Band; LVESV: Left Ventricular End-Systolic Volume; LVEDV: Left Ventricular End-Diastolic Volume; PCI: Percutaneous Coronary Intervention; MACE: Major Adverse Cardiovascular Event; AKI: Acute Kidney Injury; ACS: Acute Coronary Syndrome; LVT: Left Ventricular Thrombus; STEMI: ST-elevation myocardial infarction; AUC: Area under curve; EMPACT-MI: EMPAgliflozin for the prevention of Chronic heart failure and morTality after an acute Myocardial Infarction; E/e’: E wave to e velocity. [20-32]

Author(s)/Year	Study design/Sample Size	Baseline LVEF	Intervention details	Primary outcomes	Secondary outcomes	Findings
Carberry et al., 2025	Randomized, double-blind, placebo-controlled trial; 105 patients	Mean LVEF: 34.8 ± 6.0%	Empagliflozin 10 mg once daily vs. placebo, added to standard care for 24 weeks	Change in LVESVI at 24 weeks	Changes in LVEDVI, LVEF, LAVI, LVMI, NT-proBNP, hs-TnI, infarct size, and exploratory biomarkers	No significant effect of empagliflozin on LVESVI (between-group difference: 0.3 mL/m², 95% CI: −5.2 to 5.8; p=0.92). No effect on secondary outcomes. Progressive adverse remodelling did not occur in most patients.
Butler et al., 2024	Randomized, double-blind, placebo-controlled trial; 6,522 patients (3,260 empagliflozin, 3,262 placebo)	78.4% had LVEF<45%; 20.6% had LVEF≥45% among those with congestion	Empagliflozin 10 mg daily or placebo within 14 days post-MI, added to standard care	Composite of first hospitalization for HF or death from any cause (HR 0.90; 95% CI 0.76–1.06; P=0.21)	Total HF hospitalizations/death (rate ratio 0.87; 95% CI 0.68–1.10); individual HF hospitalization (HR 0.77; 95% CI 0.60–0.98)	No significant reduction in primary outcome. Lower HF hospitalization risk (HR 0.77; 95% CI 0.60–0.98). Death rates similar (HR 0.96; 95% CI 0.78–1.19).
Jolly et al., 2025	Randomized, double-blind, placebo-controlled trial (2x2 factorial); 7,062 patients (3,528 colchicine, 3,534 placebo)	Not explicitly reported (enriched for NSTEMI with LVEF ≤45% or other risk factors)	Colchicine 0.5 mg daily (initially weight-based, then uniform) or placebo, started early post-MI for median 3 years	Composite of CV death, MI, stroke, or ischemia-driven revascularization (HR 0.99; 95% CI 0.85–1.16; P=0.93)	CV death/MI/stroke (HR 0.98; 95% CI 0.82–1.17); total recurrent events (HR 0.98; 95% CI 0.85–1.13)	No benefit: Primary outcome similar between groups. Lower CRP with colchicine (MD −1.28 mg/L; 95% CI −1.81 to −0.75). Increased diarrhea (10.2% vs. 6.6%; P<0.001).
Tong et al., 2020	Double-blind, randomized, placebo-controlled trial (151 patients; 60 in MRI substudy)	Mean LVEF: 44% (placebo), 45% (colchicine)	Colchicine (2-mg loading dose, then 0.5 mg twice daily for 5 days) vs. placebo	Area under the curve of CK-MB over 72 hours	MRI-LGE infarct size, LVEF, inflammatory markers	Primary outcome: Lower CK-MB AUC in colchicine group (3144 vs. 6184 ng·h·mL⁻¹, P<0.001); MRI subgroup: Smaller infarct size (18.3 vs. 23.2 mL/1.73 m², P=0.019) and relative infarct size (13.0% vs. 19.8%, P=0.034); Higher predischarge LVEF (53% vs. 46%, P=0.003); Reduced inflammatory markers (neutrophils: P=0.008; CRP: P=0.019).
von Lewinski et al., 2022	Multicentre, double-blind RCT; 476 patients (237 empagliflozin, 239 placebo)	Median: 48% (IQR: 43–53) in empagliflozin group; 49% (IQR: 43–54) in placebo group	Empagliflozin 10 mg/day or placebo initiated within 72 hours post-PCI for acute MI (creatine kinase >800 U/L).	Change in NT-proBNP levels over 26 weeks.	Changes in LVEF, E/e’, LVESV, LVEDV, ketone bodies, hospitalizations, and safety outcomes.	NT-proBNP: 15% greater reduction with empagliflozin (95% CI: -4.4% to -23.6%; *p=0.026); LVEF: Absolute improvement of 1.5% (95% CI: 0.2% to 2.9%; *p=0.029). LVESV/LVEDV: Reduced by 7.5 mL (*p=0.0003) and 9.7 mL (*p=0.0015), respectively.
Tardif et al., 2019	Double-blind, randomized, placebo-controlled trial; 4,745 patients with recent MI (<30 days)	Excluded if LVEF <35% (mean LVEF not reported)	Colchicine 0.5 mg daily vs. placebo	Composite of CV death, cardiac arrest, MI, stroke, or urgent angina-related revascularization	Components of primary endpoint, total mortality, atrial fibrillation, biomarker changes	Colchicine reduced primary endpoint by 23% (HR 0.77, 95% CI 0.61–0.96, P=0.02), driven by fewer strokes (HR 0.26, 95% CI 0.10–0.70) and urgent revascularizations (HR 0.50, 95% CI 0.31–0.81). No significant effect on CV death (HR 0.84, 95% CI 0.46–1.52).
Bouleti et al., 2024	Randomized controlled trial/192 patients	Mean LVEF: 45% (colchicine), 44% (placebo)	Colchicine (2 mg loading dose + 0.5 mg twice daily for 5 days) vs. placebo, initiated at reperfusion for STEMI	Composite MACE (all-cause death, ACS, HF events, ischemic strokes, ventricular arrhythmias, AKI) at 1 year	Quality of life (EQ-5D score), drug therapy prescriptions, LVT incidence	No significant difference in MACE at 1 year (35.6% colchicine vs. 44.1% placebo, p=0.3). Trend toward fewer HF events with colchicine (11.9% vs. 19.8%, p=0.20). No difference in ischemic strokes despite higher LVT with colchicine (22.2% vs. 7.4%, p=0.01).
Karim et al., 2025	Randomized, double-blind, placebo-controlled trial; 77 patients (37 colchicine, 40 placebo)	78.4% had LVEF <45%	Colchicine 2 mg loading dose + 0.5 mg every 12h for 2 days vs. placebo, initiated peri-PPCI	Reperfusion injury (RI): composite of low-flow TIMI 0–2, arrhythmia, cardiogenic shock, or persistent chest pain	Adverse events (e.g., diarrhea, GI bleeding)	No benefit: RI incidence similar (51.5% vs. 42.4%; P=0.437). Higher diarrhea with colchicine (16% vs. 7.5%; P not reported).
Defteros et al., 2015	Randomized controlled trial/151 patients (60 in MRI substudy)	Newly developed LVEF <45%	Colchicine vs. placebo for 9 days post-STEMI; initiated within 12 hours of symptom onset; all patients received PCI	CK-MB	infarct size, neutrophil count	AUC CK-MB was significantly lower in colchicine group (p<0.001); infarct size (MRI-LGE) was lower (18.3 (7.6–29.9) vs. 23.2 (18.5–33.4) mL/1.73 m², p=0.019); relative infarct size reduced (13.0% vs. 19.8%, p=0.034); neutrophil count lower (7543 vs. 8922/μL, p=0.008); all correlated with infarct size and CK-MB (p=0.001)
Hernandez et al., 2024	Double-blind, randomized, placebo-controlled trial; 6,522 patients with acute myocardial infarction	Newly developed LVEF <45% or signs/symptoms of congestion	Empagliflozin 10 mg daily vs. placebo within 14 days of admission	Composite of time to first hospitalization for HF or all-cause death (not significant)	First HF hospitalization, total HF hospitalizations, HF adverse events, new HF therapies	Empagliflozin reduced first HF hospitalization by 23% (HR 0.77, 95% CI 0.60–0.98, P=0.031) and total HF hospitalizations by 33% (RR 0.67, 95% CI 0.51–0.89, P=0.006). Benefit consistent across subgroups.
Udell et al., 2024	Randomized controlled trial (EMPACT-MI)/6,522 patients	Mean LVEF: 41 ± 9%	Empagliflozin (10 mg daily) vs. placebo, initiated within 14 days of acute MI	Time to first HF hospitalization or all-cause mortality (HR: 0.90; 95% CI: 0.76–1.06; P = NS)	Total HF hospitalizations, HF adverse events, safety outcomes (e.g., hypotension, AKI)	Empagliflozin reduced first HF hospitalization by 23% (HR: 0.77; 95% CI: 0.60–0.98) and total HF hospitalizations by 33% (RR: 0.67; 95% CI: 0.50–0.89); No effect on mortality. Benefits were consistent across LVEF subgroups (all P for interaction ≥0.79).
Sourij et al., 2024	Randomized, double-blind, placebo-controlled trial; 476 patients (84 women, 392 men)	Median LVEF: Women: 49% (IQR 45–55%), Men: 48% (IQR 42–53%)	Empagliflozin 10 mg once daily vs. placebo, initiated ≤72h post-PCI for 26 weeks	Changes in NT-proBNP levels at 26 weeks	LVEF, LVESV, LVEDV, E/e′; sex-stratified analysis	Empagliflozin reduced NT-proBNP similarly in women and men (adjusted reduction: 15%, 95% CI: −23.6% to −4.4%; P=0.984). Improved LVEF (women: +5.7%, men: +4.5%) and reduced LV volumes (P> 0.05 for all).
Mewton et al., 2021	Double-blind, randomized, placebo-controlled trial (192 patients)	Newly developed LVEF <45%	Colchicine (2 mg loading dose, then 0.5 mg twice daily for 5 days) vs. placebo	Infarct size (IS) at 5 days by cardiac MRI	LV remodeling, LV thrombus incidence, LVEF at 5 days and 3 months	No significant reduction in IS (26.0 g vs. 28.4 g, P=0.87). No difference in LV remodeling (+2.4% vs. -1.1%, P=0.49) or LVEF (46% vs. 44%, P=0.06). Higher LV thrombus incidence with colchicine

Empagliflozin in Post-STEMI Patients with Reduced LVEF

Efficacy on left ventricular (LV) remodeling and function: There are a number of studies that estimated the effects of empagliflozin on the LV structure and biomarkers after the STEMI among patients with low ejection fraction. In the Carberry et al. trial (mean LVEF 34.8%), empagliflozin did not significantly change the primary outcome of LV end-systolic volume index (LVESVI), an intragroup difference of 0.3 mL/m^2^ was obtained (p=0.92). Other secondary outcomes including LVEF and cardiac markers were also not affected [20]. Nevertheless, better results were described by von Lewinski et al. with the median LVEF of 48-49% and empagliflozin resulting in a 15% relative decrease in N-terminal pro-B-type natriuretic peptide (NT-proBNP) levels (p=0.026) and an absolute increase in the LVEF by 1.5% (p=0.029) [24]. In line with these observations, Sourij et al. reported that empagliflozin significantly increased LVEF in both sexes, by 5.7% among women and 4.5% in men and had an 15% adjusted median change in NT-proBNP (-23.6 percent to -4.4 percent) [31]. Such findings imply that empagliflozin improves LV and cardiac biomarkers, with moderate and convergent effects on parameters such as NT-proBNP and LV volumes, independent of a variety of patient subsets.

HF hospitalization and mortality: Empagliflozin has always shown benefit in minimizing HF hospital re-admission following STEMI in large-scale trials. The EMPAgliflozin for the prevention of Chronic heart failure and morTality after an acute Myocardial Infarction (EMPACT-MI) trial with Butler et al. did not indicate substantial lowering of the composite primary outcome of HF hospitalizations or all-cause mortality (HR 0.90; p=0.21), but indicated a significant decrease in first HF hospitalizations (HR 0.77; 95% CI: 0.60-0.98) [21]. Hernandez et al. also showed a 23% relative decrease in first HF hospitalization (p=0.031) and 33% relative decrease in overall HF hospitalization (p=0.006) as well [29]. These findings were confirmed by Udell et al. who further confirmed the use of empagliflozin in reducing HF-related events, though again showing no significant result on mortality [30]. In combination, these results are consistent in the finding that empagliflozin would prevent HF decompensation in post-MI patients with a reduced LVEF although it is seen to be neutral in the outcome of survival.

Colchicine in Post-STEMI Patients with Reduced LVEF

Effect on inflammation and LV remodeling: Colchicine is a potent anti-inflammatory agent that may influence post-infarction healing and ventricular remodeling. In a study by Tong et al., the infarct size and inflammation were considerably lowering with colchicine in a group (a reduced Creatine Kinase-Myocardial Band (CK-MB) area under the curve, p<0.001) and a higher pre-discharge LVEF (53% vs. 46%, p=0.003) [23]. Conversely, Mewton et al. showed that early administration of colchicine following MI did not alter the infarct size, LV remodeling, and LVEF in a statistically significant way [32]. Meanwhile in the Colchicine Cardiovascular Outcomes Trial (COLCOT) trial by Tardif et al., there was a 23% reduction in the composite cardiovascular outcomes (p=0.02) in the colchicine group relative to placebo, mostly due to stroke volume depreciation and urgent revascularizations, not LVEF [25]. These reports indicate that although colchicine can effectively treat inflammation and alleviate ischemic complications, its impact on the processes of ventricular remodeling, as well as LVEF, cannot be considered uniform and, it seems, less pronounced when compared to empagliflozin.

Cardiovascular outcomes and safety: The effects of colchicine on clinical outcomes and safety have been contradicting. When colchicine was compared to placebo in the Colchicine and Spironolactone in Patients with Myocardial Infarction (CLEAR) trial by Jolly et al., it was found that colchicine failed to alleviate the risk of major adverse cardiovascular events (MACE) (HR 0.99; p=0.93), but it resulted in a decreased level of CRP. The rate of gastrointestinal adverse events, especially diarrhea was much higher in the colchicine group (10.2% vs. 6.6%; p<0.001) [22]. On the one hand, Karim et al. did not find any advantage of colchicine in the prevention of reperfusion injury but revealed an increased rate of gastrointestinal side effects [27]. Bouleti et al. have reported that the one-year MACE was equally distributed between colchicine and placebo groups; although, there was a non-significant trend of fewer HF events (11.9% vs. 19.8%; p=0.20). Of note, the incidence of LV thrombus was also significantly increased in this trial with colchicine (22.2% vs. 7.4%; p=0.01) [26]. These findings suggest that in patients with HF, despite the potential role of colchicine in decreasing the incidence of selected ischemic events such as stroke and revascularization, the role of colchicine in HF prevention has not been elucidated, and its application is hampered by tolerability issues and thrombotic risk.

Comparative Interpretation

Empagliflozin and colchicine show notable differences in efficacy, mechanisms, and safety among post-STEMI patients with reduced LVEF. Empagliflozin consistently improved LVEF across trials by von Lewinski et al. [24] and Sourij et al. [31], with gains between 1.5-5.7%. In contrast, colchicine showed inconsistent results: Tong et al. [23] reported improved pre-discharge LVEF, while Mewton et al. [32] and Tardif et al. [25] found no change. Empagliflozin also reduced NT-proBNP, a marker of cardiac stress, by around 15% in multiple studies. Colchicine trials paid less attention to NT-proBNP but showed reductions in inflammatory markers such as CRP and CK-MB [22,23].

In terms of outcomes, empagliflozin reduced HF hospitalizations with hazard ratios of 0.77-0.87 [21,29,30]. Colchicine, however, did not lower HF hospitalizations or MACE in most trials, although stroke risk was reduced in the COLCOT trial [25]. Safety profiles also differed. Empagliflozin was generally well tolerated, with no major adverse effects reported. Colchicine, however, was associated with gastrointestinal side effects, particularly diarrhea, and a higher incidence of LV thrombus formation in STEMI patients. From a health economics perspective, colchicine demonstrated cost-effectiveness in both short- and long-term models.

Discussion

This systematic review set out to evaluate the effects of empagliflozin and colchicine in patients with reduced LVEF following STEMI, and the findings align well with this objective. Empagliflozin demonstrated consistent improvements in surrogate markers of cardiac function, including modest gains in LVEF and significant reductions in NT-proBNP, as seen in von Lewinski et al. [24] and Sourij et al. [31]. These results support the objective by highlighting its positive role in ventricular remodeling and myocardial stress reduction. The evidence also confirms empagliflozin’s ability to reduce HF hospitalizations, as consistently reported across EMPACT-MI [21] and subsequent analyses, thereby meeting the review’s aim of assessing clinical outcomes.

In contrast, colchicine showed variable effects, with benefits largely confined to reductions in inflammatory markers (e.g., CRP) and ischemic events such as stroke in COLCOT, but inconsistent findings regarding LVEF and LV remodeling. This aligns with the review’s objective by demonstrating that while colchicine may have anti-inflammatory and ischemic benefits, its role in improving functional cardiac outcomes remains inconclusive. By synthesizing results across efficacy, mechanism, and clinical outcomes, the review provided a balanced comparative interpretation. The consistency of empagliflozin’s effects and the variability of colchicine’s outcomes directly address the stated objective of clarifying the therapeutic potential of these agents in post-STEMI patients with reduced LVEF.

Carberry et al. confirmed that empagliflozin had a consistent effect on the surrogate markers of cardiac function and improved HF hospitalizations, and colchicine produced more fluctuated effects, especially on functional outcomes of the cardiac work [20]. These findings correlate with meta-analyses by Forycka et al., which determined that empagliflozin resulted in decreased HF hospitalization (by 33%) with no impact on mortality outcome, and did show modest benefit on LVEF, making our findings appear consistent with the available larger body of evidence [33].

Empagliflozin has shown small, steady effects on heart-remodeling variables in clinical studies. In other research studies like von Lewinski et al. [24] and Sourij et al. [31]. LVEF improved by 1.5-5.7%, and major reductions in NT-proBNP levels were observed. These results indicate a positive outcome on ventricular load and myocardial stress. These findings are consistent with judgments drawn by other systematic reviews, including a pooled investigations of SGLT2 inhibitors in cardiovascular pathologies by Madonna et al., which showed that empagliflozin has unrivaled effects on heart structure and performance, especially in individuals with fresh cardiovascular accident and low ejection fraction [34].

Compared to the clinical outcomes, empagliflozin always showed a reduction in the number of HF-related hospitalizations in post-STEMI patients. The EMPACT-MI trial by Butler et al. as well as later analyses by Hernandez et al. and Udell et al. demonstrated these results to be true of the first and overall HF hospitalizations too [21,29,30]. These conclusions are supported by the previous meta-analyses by Usman et al., which combined SGLT2 inhibitor data in acute and chronic HF conditions and determined the applicability of empagliflozin in the prevention of HF decompensation and HF progression [35]. This suggests that while empagliflozin effectively prevents HF events, its effect on overall survival is limited.

Though mechanistically different than empagliflozin, colchicine may have a beneficial effect due to its anti-inflammatory properties. Multiple trials, such as a study by Tong et al. and the COLCOT trial by Tardif et al., reported a decrease in the inflammatory marker CRP and ischemic events like stroke [23,25]. The results were conflicting in terms of LVEF and LV remodeling and contradictory for MACE. This is consistent with Younas et al., who reported that the effects of colchicine in patients with MI were promising but inconclusive because of inconsistent trial design, endpoints, and selection criteria of the patients [36].

Clinical implications

The evidence provides a real base to use empagliflozin in patients with low LVEF after STEMI, especially to prevent hospitalization due to HF as well as possible reduction of adverse ventricular remodeling. The concomitant risks, long-term safety record, and adaptability to customary post-MI treatment make it worthwhile for the management of this risky group. Colchicine an equally promising anti-inflammatory agent that potentially decreases the subsequent ischemic events like stroke, has more inconsistent clinical findings. Its application can be discussed in patients with raised inflammatory markers or unrelenting ischemic risk, yet clinicians have to carefully estimate the danger of gastrointestinal adverse effects and the probability of thrombotic issues. Significantly, the affordability of colchicine means that it has a potential role to play in resource-limited environments, but its systematic administration in all post-STEMI patients cannot be advocated.

Limitations

This review has several important limitations. First, there was notable heterogeneity in how LVEF was defined, measured, and reported across trials. Some studies reported mean values, while others used medians and interquartile ranges, making direct comparisons challenging. Second, the study populations were not entirely homogeneous, as the timing of drug administration after MI varied considerably, with some trials initiating treatment immediately post-STEMI and others delaying initiation until later phases of recovery. This variability may have influenced the observed effects on cardiac remodeling and functional outcomes. Third, the included trials differed widely in sample size. Several studies were relatively small and potentially underpowered to detect clinically meaningful differences, particularly in hard outcomes such as mortality and HF hospitalization. These issues introduce variability and limit the strength of pooled interpretations, underscoring the need for larger, well-designed RCTs with standardized endpoints to confirm the comparative efficacy of empagliflozin and colchicine in this setting.

## Conclusions

Empagliflozin appears to be a consistently beneficial option in reducing HF-related hospitalizations and improving cardiac function in patients with reduced ejection fraction after MI. However, these findings should be interpreted cautiously, as most evidence comes from short- to medium-term trials, and long-term outcomes, including mortality, remain less certain. Colchicine may provide additional benefits through its anti-inflammatory and anti-ischemic properties, but its effects on functional cardiac outcomes and major cardiovascular events are inconsistent across studies. Moreover, safety concerns, including gastrointestinal intolerance and reports of increased risk of ventricular thrombus formation, warrant careful consideration. Patient selection, timing of treatment initiation, and heterogeneity in trial design likely contribute to these differences, underscoring the need for further large-scale, well-powered studies before either treatment can be prioritized in post-STEMI care.
